# Health Service Access among Indonesian Migrant Domestic Workers in Taiwan

**DOI:** 10.3390/ijerph18073759

**Published:** 2021-04-04

**Authors:** Shuen-Fu Weng, Azis Malik, Utoomporn Wongsin, Franziska Michaela Lohmeyer, Li-Fong Lin, Suleman Atique, Wen-Shan Jian, Yuherina Gusman, Usman Iqbal

**Affiliations:** 1 Division of Endocrinology and Metabolism, Department of Internal Medicine, Taipei Medical University Hospital, Taipei City 110, Taiwan; sfweng@ntu.edu.tw; 2 Division of Endocrinology and Metabolism, Department of Internal Medicine, School of Medicine, College of Medicine, Taipei Medical University, Taipei City 110, Taiwan; 3 Master Program in Global Health & Development Department, College of Public Health, Taipei Medical University, Taipei City 110, Taiwan; azismalik99@gmail.com; 4 PhD Program in Global Health & Health Security Department, College of Public Health, Taipei Medical University, Taipei City 110, Taiwan; d537108007@tmu.edu.tw; 5 Scientific Directorate, Fondazione Policlinico Universitario A. Gemelli IRCCS, 00168 Rome, Italy; franziska1.lohmeyer@gmail.com; 6 School of Gerontology Health Management, College of Nursing, Taipei Medical University, Taipei City 110, Taiwan; fong930@tmu.edu.tw (L.-F.L.); jj@tmu.edu.tw (W.-S.J.); 7 Department of Physical Medicine and Rehabilitation, Shuang Ho Hospital, Taipei Medical University, New Taipei City 23561, Taiwan; 8 Neuroscience Research Center, Taipei Medical University, Taipei City 110, Taiwan; 9 Research Center for Artificial Intelligence in Medicine, Taipei Medical University, Taipei City 110, Taiwan; 10 Department of Health Informatics, College of Public Health and Health Informatics, University of Ha’il, Ha’il City 55211, Saudi Arabia; gcufpharmd@yahoo.com; 11 School of Health Care Administration, Taipei Medical University, Taipei City 110, Taiwan; 12 International Center for Health Information Technology (ICHIT), Taipei Medical University, Taipei City 110, Taiwan; 13 International Doctoral Program for Asia Pacific Studies, National Chengchi University, Taipei City 11605, Taiwan; yuherina.gusman@gmail.com

**Keywords:** healthcare, health service access, migrant workers, global health, public health, Taiwan

## Abstract

The number of migrant workers in Taiwan increases annually. The majority is from Indonesia and most of them are female caregivers. This study aims to determine the access to health services and the associated factors among Indonesian female domestic workers in Taiwan. In this cross-sectional study, data were collected from February to May 2019, using a structured questionnaire. Subsequently, multiple logistic regression was used to examine the association between socio-demographic factors and health service access. Two hundred and eighty-four domestic migrant workers were interviewed. Eighty-five percent of the respondents declared sickness at work, but only 48.8% seek health care services. Factors associated with health service access were marital status, income, and the availability of an attendant to accompany the migrant workers to the healthcare facilities. Language barrier and time flexibility were the main obstacles. Further research and an effective health service policy are needed for the domestic migrant workers to better access health care services.

## 1. Introduction

In 2018, approximately 232 million migrant workers, representing 3.1 percent of the global population, transferred to other countries or regions to seek mostly seasonal or temporary employment [1]. Taiwan is one of the most popular destinations for migrant workers from South East Asian countries. As of April 2019, 706,060 migrant workers actively worked in Taiwan: 38.4% from Indonesia, 31.4% from Vietnam, 21.8% from the Philippines, and 8.4% from Thailand and other countries [2]. Migrant workers are one of the most vulnerable groups within society, as they often perform so-called 3D jobs, which means dirty, dangerous, and demanding, working longer hours with less pay. Additionally, they often experience violence and exploitation.

Part of this group [3] (globally around 11.5 million or 7.7% of all migrant workers), specifically vulnerable to abuse, illness, and mental health problems, are migrant domestic workers, who perform work in or for households. The role of these migrant domestic workers, of which 74% are women, is significant for the economy in both sending and receiving countries [3]. However, they are often hidden from or invisible to the public eye and public policy and have limited access to medical care [4].

As a health service is recognized as a human right under the international regulation of the International Covenant on Economic, Social, and Cultural Rights (ICESCR) and migrant domestic workers are a specific group requiring protection, sending and recipient countries should adopt standards or specific procedures to guarantee access to health services and to promote their health. Sufficient data concerning health services for migrant domestic workers are needed for policymakers and stakeholders to develop appropriate public health strategies and to arrange preventive actions.

Moreover, the health status of migrant workers is not only necessary for themselves but also for employers and their families. Research showed the important role of the health status of foreign domestic workers to reduce the negative impacts of caregiving on families [5]. Despite this, studies conducted in Malaysia [6], Hong Kong [7], Singapore [8], and Thailand [9] concluded that health service access of migrant workers, and specifically of migrant domestic workers, is very low. The main problems are discrimination, health service fees (which are higher for migrant workers than for locals), time flexibility, language barrier, and lack of knowledge.

This study assessed health service access of Indonesian migrant domestic workers in Taiwan and compared the results obtained with those of previously conducted studies in other countries.

## 2. Materials and Methods

This cross-sectional study was conducted from February to May 2019 in Taipei, Taiwan, one of the areas with the highest number of Indonesian migrant domestic workers, and recruited study participants in public areas of the city center. Informed consent was obtained from each participant before questionnaire administration. Approval to conduct this study was sought from the TMU Joint Institutional Review Board (Certificate of TMU-JIRB No.: N201812037).

### 2.1. Sample Size Calculation and Inclusion Criteria

The sample size was calculated considering a medium effect size (0.15) with alpha equal to 0.05 and 14 (demographic) predictors using G Power version 3.1 software (Heinrich-Heine-Universität, Düsseldorf, Germany). Two hundred twenty-three participants were estimated, and 250 questionnaires were distributed. Inclusion criteria were Indonesian citizenship, migrant domestic worker status, ability to speak, read, and write Indonesian, health facility access as a patient or caregiver in the past, female gender, and age ≥ 20 years.

Health facility access was defined as the participants’ access to a health facility as a patient or caregiver accompanying other persons in need of health care.

### 2.2. Data Collection

Data were collected through a self-administered and structured questionnaire previously used for another study with a similar purpose [9] with some adjustments related to the conditions in Taiwan. Five experts in the field were consulted and 30 Indonesian migrant domestic workers were recruited to further check the reliability and level of understanding of the questions.

The questionnaire was divided into two sections: the first was related to demographic characteristics of the participants (14 questions); the second was related to health service access (24 questions) using a five-item Likert-scale (strongly disagree, disagree, neutral, agree, strongly agree). We pre-tested the questionnaire for internal reliability, measured by calculating Cronbach’s alpha, which was >0.7.

### 2.3. Study Variables

The independent variables were the demographic characteristic of the respondents (Table 1). The dependent variables were the five domains of health service access: availability, accessibility, accommodation, affordability, and acceptability as defined by Penschansky and Thomas. The authors developed the 5A concept of access in the context of health services arguing that access to health services is complex and specific and should not only focus on the use of health services [10]. Definitions of the five domains are stated in Table 1. Based on the scores obtained from each of the five above-mentioned dependent variables, we defined a further dependent variable, which summarizes health care access. Adding up all the scores and dividing them by the number of participants, we obtained a mean value used as a cut-off point. Participants with scores above this mean were regarded as having a high healthcare access while participants with scores below this mean were regarded as having low healthcare access.

### 2.4. Data Analysis

Descriptive statistics of demographic data and access to health services were analyzed as standard deviation (SD), number (*n*), and percentage (%) as appropriate. In order to gain insights on the different domains of the 5A concept of access theory for each of the five dimensions, a mean was calculated by adding up all scores and dividing them by their count. Odds ratios (ORs) with a 95% confidence interval (Cl) and *p*-values were reported to measure the probability to have a high access to health services. The Chi-square test was used to compare the difference in the distribution between those with high and those with low health service access. Logistic regression analysis was used to examine the relationship between demographic characteristics and health service access among Indonesian domestic migrant workers. All statistic tests were two-sided and the significance level was set at *p* < 0.05. Data analysis was performed with SPSS (Statistical Package for Social Science) version 20 for Windows (SPSS, Chicago, IL, USA).

## 3. Results

### 3.1. Socio-Demographic Characteristics of Participants

Two hundred and forty-eight Indonesian female migrant domestic workers were surveyed, of which almost a third were aged between 36 and 40 years. Most migrant workers were married and graduated from senior high school. Most workers had been staying in Taiwan for more than three years and 89.1% worked more than 11 h per day. Only 0.8% of them worked less than 5 h a day. Their monthly income was between NTD 16,000–20,000 (USD 514–643 approx.). Many women worked as caregivers and lived with their employer. All participants had national health insurance (100%) and 85.1% of them experienced sickness during their stay in Taiwan and accessed as patients the Taiwanese national health system. Those who lived near medical clinics were 44.8%, and for 79.4% the distance to health services was less than 5 km. For 60.9% of women, an attendant was available to accompany them to access the health facility (Table 2).

### 3.2. Distribution Score of the Five Domains Access to Health Services

Acceptability had the highest mean (20.54) followed by affordability (19.37), and availability had the lowest mean (14.59) (Supplementary Table S1).

#### 3.2.1. Availability

Regarding availability, 28.6% of the respondents strongly agreed and 41.9% agreed that services are not provided in Indonesian and doctors who speak Indonesian are not available. Instead, 29.4% strongly agreed and 39.9% agreed that translators are not provided (Table S2 in Supplementary Materials).

#### 3.2.2. Accessibility

Overall, accessibility to health services was satisfying; 61.7% agreed that health facilities are easy to access, 58.1% answered that no problem with health service cost/payment exists, 69.8% agreed that health facilities are close to their place of living, and 56% replied that the ambulance is always available in the case of emergencies. However, 8.5% of the respondents had problems paying for the services provided, and 15.7% remained neutral (Table S3 in Supplementary Materials).

#### 3.2.3. Accommodation

About 62% of workers answered feeling comfortable with health services and 47.6% received health service guidelines in the past, even though they cannot read Chinese. Instead, 43.1% of women replied that guidelines were easy to understand due to the assistance from volunteers in the hospital (41.5%). However, 25% of respondents disagreed and strongly disagreed when asked if they received help from volunteers at the hospital to gain access to care and 14.5% were neutral. In addition, 25% of them disagreed and strongly disagreed that they received expedited services during their visits to the hospital (Table S4 in Supplementary Materials).

#### 3.2.4. Affordability

More than half of the respondents (58.5%) had no difficulty accessing national health insurance and 53.2% of them already knew that they had to pay the registration fee, which they considered as inexpensive (53.6%). Moreover, 52.4% did not object to paying an extra payment if necessary. However, 21.8% of women responded neutrally regarding enough personal resources to pay out of pocket health care, while 10.1% disagreed and 3.25% strongly disagreed. Finally, 12.5% disagreed to pay out of pocket in addition to the regular copayment and registration fee (Table S6 in Supplementary Materials).

#### 3.2.5. Acceptability

The majority of women who accessed health facilities agreed that health workers in Taiwan respect patients from other countries (59.3%), pay full attention to the patients’ needs (61.3%), and assess symptoms comprehensively (55.2%). In general, 59.7% of respondents trust health workers and feel no discrimination (58.5%). Generally, the acceptability is good and quite positive regarding access to health services (Table S6 in Supplementary Materials).

#### 3.2.6. Access to Health Services

The sum of the scores obtained by each participant (22,108) was divided by the number of participants (248), obtaining a cut-off point of 89.15 to differentiate between high and low healthcare access.

### 3.3. Bivariate Analysis among Determinant Factors and Health Service Access

Migrant domestic workers older than 35 years, those who stayed in Taiwan for more than one year, and those working more than 11 h per day reported lower access to health services (OR = 0.75, OR = 0.7, and OR = 0.63, respectively). Married domestic migrant workers, those who graduated from senior high school, and migrant workers with an income equal or higher than NTD 16,000 had higher odds to access health services (OR = 1.88, OR = 1.283, and OR = 2.895, respectively). Migrant workers who worked as housekeepers reported higher access to health services compared to caregivers (OR = 1.283). Among 248 respondents, 211 experienced sickness, around 52.6% utilized health services, and about 59.7% choose a public hospital. About 79.4% lived not far from a healthcare facility (<5 km) and these migrant workers reported higher access to health services compared with migrant workers living 6–10 km from a healthcare facility (OR = 1.843). Migrant workers who were accompanied by employers’ staff (60.8%) also observed higher access to the health facilities (OR = 1.87) compared with unaccompanied migrant domestic workers (Table 3).

### 3.4. Multivariable Analysis for Factors Associated with Health Service Access of Migrant Workers

Three variables were positively associated with health service access in the univariate and multivariate analysis namely, marital status (AOR = 2.007; 95% CI: 1.175–3.429), income (AOR = 2.916; 95% CI: 1.302–6.527), and the availability of an attendant to accompany the respondent to health facilities (AOR = 1.720; 95% CI: 1.011–2.927) (Table 4).

## 4. Discussion

Comparing our results with data published in the literature, we found that Indonesian migrant domestic workers in Taiwan had higher access to healthcare facilities than those in other countries. Previous research, particularly in Hong Kong, Malaysia Singapore, and Thailand [6,7,8,9], stated that health service access for migrant workers is low. In this study, the main obstacles regarded discrimination, higher health service fees compared with those of locals, time flexibility, language barrier, and lack of knowledge. Instead, in Taiwan, obstacles were correlated to language barriers and time flexibility, which we demonstrated with the 5A concept of access theory (Table 1) [10].

More detailed, regarding availability of health services and language barriers, which was one of the main concerns for Indonesian domestic migrant workers, the availability of doctors or translators speaking Indonesian during checkups and hospital visits was low. This is similar to the situation in Hong Kong, where most of the workers came from South East Asia, but the official language for medical services is Chinese and English.

Regarding accessibility, most of the respondents agree and strongly agree that health facilities are easily accessible. This was related to short distances from home to hospitals. Furthermore, the respondents had no problem with payment for health services, but time flexibility was negatively judged. Similar to the situation in Hong Kong, most of the health services are available during weekdays; however, migrant workers have their free day on only Saturdays or Sundays, which hampers the access to health services for check-up visits [11]. However, access of migrant workers in Taiwan is worse than in Hong Kong, because in Taiwan, caregivers or housekeepers have only one day off per month [12,13,14], which decreases the time flexibility to access the health services in our study even further.

The accommodation to access health services for Indonesias domestic migrant workers was satisfying. However, the percentage of respondents who answered neutral, disagree, and strongly disagree about having ever received expedited services during hospital visits and assistance from a volunteer was also quite high. This might have occurred because most of them preferred a medical clinic rather than a hospital, where expedited services and volunteer support are unavailable. A similar situation was demonstrated in Singapore, where accommodation to access health services for migrant workers improved after some NGOs educated migrant workers about their rights and obligations as temporary workers [8].

Regarding affordability, 100% of the respondents were covered by Taiwanese national health insurance, which is a single-payer, compulsory social insurance system with centralized disbursement of healthcare funds financed through payroll tax paid premiums. Consequently, NTD 290–310 (US$ 9.32–9.97 approx.) are automatically deducted from their salary per month to pay insurance bills, while the employer pays the rest. About 58.5% of the respondents agree to have no difficulties accessing health insurance services, which was a better result compared with other recipient countries such as Indonesia, Hong Kong, and Singapore. However, health care insurance and knowledge regarding access have no positive influence on access to health services. One hypothesis is that, even if the workers have health insurance, they do not access the health service out of fear to be repatriated if their employer knows that the worker is sick. Therefore, workers did not use health services despite health insurance and favorable distance from their living place to the health services center. This is in contrast to another study, which found that the most common reasons for non-utilization of health services were lack of transportation and lack of knowledge on where to seek care [15]. Migrant workers in Hong Kong, Malaysia, Thailand, and Singapore encountered problems regarding health service costs, which led to less utilization of health services. In Hong Kong, most migrant workers could not afford the consultation fee [7]. In Thailand, workers could not afford health service costs due to low income [9]. Instead, Malaysian migrant workers could not afford medical treatment costs because they had to pay more than local residents [6], and in Singapore, the removal of subsidies for migrant workers in 2007 caused health care service access problems [8]. However, in Taiwan, most respondents agreed and strongly agreed that health services cost is affordable and 69.3% stated that they could afford extra payment such as medicine and other services. However, 35.1% of the respondents, with salaries higher than NTD 16,000, were reluctant to pay out of pocket for medical treatment. The answers might be different for those seriously ill staying in the hospital since their job is temporarily terminated and they cannot receive their salary; many of them need to rely on family financial support from Indonesia and charities collected by the Indonesian migrant worker associations in Taiwan.

Compared with the other five domains, acceptability had the highest score, which leads to conclude that migrant workers in Taiwan are satisfied with the treatment received and attitudes of health workers. The respondents did not feel discriminated against during health service access. This situation is quite different from Malaysia and Hong Kong [6,7] where migrant workers face discrimination; one example, already mentioned above, is that they have to pay more for treatment and medicines compared with locals. In Taiwan, migrant workers agree that service costs are the same for migrant workers and the Taiwanese. Additionally, discrimination occurs among undocumented migrant workers because they are required to show their passports to access health services. Taiwan has similar regulations as Malaysia and the health service cannot be provided to undocumented migrant workers. In Hong Kong, many migrant workers do not access the health service because they do not feel comfortable with the service provided. Moreover, they consider the communication used by the health workers in Hong Kong as not patient-oriented [11].

Among our limitations, <15% (37) of respondents accessed healthcare facilities as caregivers and not as patients. However, these respondents answered equally the questions regarding accommodation (“I feel comfortable receiving health services” and accessibility “I do not have problems of being unable to pay for the services provided”), which is supposed to require personal healthcare facility access to be answered. Nevertheless, given the nature of their job, they had experience of healthcare facility access in the past and were, therefore, not excluded from our data analysis. Further studies from different countries are recommended to identify health service access gaps, which would improve the generalizability of the study results and give insights on health service access of different regions across the globe.

## 5. Conclusions

Health services access data are needed for government agencies to implement adequate public health policies for domestic migrant workers.

Our results demonstrated that the access of migrant workers to health services in Taiwan is higher or good compared with other recipient countries of migrant workers. Among the advantages, Taiwan has affordable health care costs compared with other Asian countries. However, aspects for improvement regard language barriers and time flexibility. An effective health service policy should be formulated for domestic migrant workers in Taiwan since health service access is recognized as a human right and migrant workers are a vulnerable group requiring support to assure health service access to promote their health status.

## Figures and Tables

**Table 1:** The implementation of the 5A concept of access theory in Taiwan.

Concept	Description	Condition in Taiwan
Availability	The relationship between volume and type of existing services (and resources) and clients’ volume and types of needs.	Health promotion: agree (53.2%) Specialist doctor: agree (58.9%) Availability of Indonesian Language services: disagree (41.9%) Availability of doctor speaking Indonesian: disagree (41.9%) Availability of translator: disagree (39.9%)
Accessibility	The relationship between the location of supply and the location of clients, taking account of client’s transportation resources regarding travel time, distance, and travel costs to health facilities.	Easy travel to health facilities: agree (61.7%) Ability to pay services: agree (58.1%) Short Distance to health facility: agree (69.8%) Easy access to ambulance: agree (56%)
Accommodation	The relationship between the manner in which the supplied resources are organized to accept clients, clients’ ability to accommodate these factors, and client’s perception of resource appropriateness.	Priority services easily accessible: agree (33.1%) Volunteer services easily accessible: agree (41.5%) Comfortability receiving health services: agree (62%) Health service guideline easily accessible: agree (47.6%) Understandability of guidelines: agree (43.1%)
Affordability	The relationship of service costs and providers’ insurance or deposit requirements in relation to clients’ income, ability to pay, and existing health insurance.	No difficulties to access national health insurance: agree (58.5%) Self-paid registration fee: agree (53.2%) Enough financial resources to pay the registration fee: agree (41.1%) Service fee affordability: agree (53.6%) Capability to pay the extra payment for medicine and other services: agree (52.4%)
Acceptability	The relationship of clients’ attitudes about personal and providers’ characteristics to real provider characteristics, as well as to provider attitudes about acceptable personal characteristics of clients.	Health workers respect the patient: agree (59.3%) Health workers pay attention to the patient’s needs: agree (61.3%) The physician pays attention to the patient’s condition: agree (55.2%) Patient trusts the health workers: agree (59.7%) Equal services compared with Taiwanese citizens: agree (58.9%)

**Table 2:** Socio-demographic characteristic of the participant (*n* = 248).

Characteristics	*n* (248)	Percent (%)
Age		
20–25 years 26–30 years 31–35 years 36–40 years 41–45 years >45	32 42 56 74 39 5	12.9 16.9 22.6 29.8 15.7 2.0
Marital Status		
Married Not married Divorced Separated	154 49 35 10	62.1 19.8 14.1 4.0
Educational Level		
Elementary school Junior high school Senior high school Bachelor Master	23 72 146 6 1	9.3 29.0 58.9 2.4 0.4
Duration of stay		
<6 months 6–12 months 1 year 2 years ≥3 years	5 29 39 45 130	2.0 11.7 15.7 18.1 52.4
Work Hours per day		
<5 h 5–10 h 11–15 h 16–20 h >20 h	2 25 65 93 63	0.8 10.1 26.2 37.5 25.4
Monthly Income		
<5000 5000–10.000 11.000–15.000 16.000–20.000 >20.000	2 2 30 177 37	0.8 0.8 12.1 71.4 14.9
Work Specification		
Caregiver Housekeeper	189 59	76.2 23.8
Living Status		
Live with employer Live with family Live with friends	245 2 1	98.8 0.8 0.4
Health Insurance		
Yes No	248 0	100 0
Ever Sick		
Yes No	211 37	85.1 14.9
Other, specify	0	
Nearest Health Facility		
Medical clinic Private hospital Public hospital	111 60 77	44.8 24.2 31.0
Distance to Health Facility		
<5 km 6–10 km	197 51	79.4 20.6
Assign someone to go with to health facilities by employee		
Yes No	151 97	60.9 39.1
Yes, because (select all that apply): a. Language barrier b. Employer wants to know your illness c. Other, specify…	111 55 0	

**Table 3:** Bivariate analysis among determinant factors and health service access.

Determinant Factors	Access to Health Service		*p*-Value	Odds Ratio	95% CI
	High Access *n* (%)	Low Access *n* (%)			
Age of participants					
>35 years ≤35 years	56 (47.5) 71 (54.6)	62 (52.5) 59 (45.4)	0.260	0.751 1	0.455–1.237
Marital status					
Married Not married	88 (57.1) 39 (41.5)	66 (42.9) 55 (58.5)	0.017 *	1.880 1	1.118–3.162
Education level					
Senior high school or higher Junior high school or lower	82 (53.6) 45 (47.4)	71 (46.4) 50 (52.6)	0.340	1.283 1	0.768–2.143
Duration of stay					
1 year or more Less than 1 year	107 (50.0) 20 (58.8)	107 (50.0) 14 (41.2)	0.339	0.700 1	0.336–1.458
Work hours per day					
11 h or more Less than 11 h	112 (50.7) 15 (55.6)	109 (49.3) 12 (44.4)	0.632	0.822 1	0.368–1.836
Monthly income					
NT$16,000 or higher Less than NT$16,000	117 (54.7) 10 (29.4)	97 (45.3) 24 (70.6)	0.006 *	2.895 1	1.320–6.348
Work specification					
Housekeeper Caregiver	33 (55.9) 94 (49.7)	26 (44.1) 95 (50.3)	0.406	1.283 1	0.713–2.309
Ever sick					
Yes No	111 (52.6) 16 (43.2)	100 (47.4) 21 (56.8)	0.293	1.457 1	0.720–2.946
Nearest health facility					
Public hospital Other health facility	46(59.7) 81 (47.4)	31 (40.3) 90 (52.6)	0.071	1.649	0.956–2.845
Distance to health facility					
<5 km 6–10 km	107 (54.3) 20 (39.2)	90 (45.7) 31 (60.8)	0.055	1.843 1	0.983–3.454
Employee assigned attendant for health facility visit					
Yes No	86 (57.0) 41 (42.3)	65 (43.0) 56 (57.7)	0.024 *	1.807 1	1.079–3.027

Logistic regression analysis, *p < 0.05, OR: Odds ratio, CI: a 95% confidence interval.

**Table 4:** Factors associated with access to health services using univariate and multivariate analysis (*n* = 248).

Determinant Factors	Crude OR (95% CI)	*p*-Value	Adjusted OR (95% CI)	*p*-Value
Marital status				
Married	1.880		2.007	
Not married (ref.)	(1.118–3.162)	0.017	(1.175–3.429)	0.011
Income				
NT$16,000 or Higher	2.895		2.916	
Less than NT$16,000 (ref)	(1.320–6.348)	0.006	(1.302–6.527)	0.009
Employee assigned attendant for health facility visit				
Yes	1.807		1.720	
No (ref.)	(1.079–3.027)	0.024	(1.011–2.927)	0.046

Logistic Regression Analysis, *p* < 0.05, OR: Odds Ratio, CI: a 95% confidence interval.

## Data Availability

The data presented in this study are available on request from the corresponding author.

## Supplementary Materials

The following are available online at https://www.mdpi.com/article/10.3390/ijerph18073759/s1, Table S1: Distribution score of five domain access to health services, Table S2: Descriptive statistic for availability (*n* = 248), Table S3: Descriptive statistic for accessibility (*n* = 248), Table S4: Descriptive statistic for accommodation (*n* = 248), Table S5: Descriptive statistic for affordability, Table S6: Descriptive statistic for acceptability.
