# Supplementary material for: Health Service Access among Indonesian Migrant Domestic Workers in Taiwan
Source: Int J Environ Res Public Health. 2021 Apr 4;18(7):3759. doi: 10.3390/ijerph18073759 (PMC8038466; doi:10.3390/ijerph18073759)
Supplement: Supplementary file 1 [file ijerph-18-03759-s001.pdf]

Supplementary Materials

Table S1. Distribution score of 5 domain access to health services.

	N	Minimum	Maximum	Mean
Availability	248	5.00	25.00	14.5927
Accessibility	248	8.00	20.00	16.0847
Accommodation	248	7.00	25.00	18.5524
Affordability	248	10.00	25.00	19.3710
Acceptability	248	9.00	25.00	20.5444

Table S2. Descriptive statistic for availability (n = 248).

No	Availability	Strongly Disagree	Disagree	Neutral	Agree	Strongly Agree
**1**	Health facilities offer health promotion, prevention, and rehabilitation services	2.0%	14.9%	12.1%	53.2	17.7%
**2**	Health facilities offer specialist services (e.g., neurologist)	2.0%	8.1 %	12.9%	58.9%	18.1%
**3**	Health services are available in Indonesian	25.8%	41.9%	5.6%	17.3%	9.3%
**4**	Some doctors speak Indonesia	28.6%	41.9%	7.3%	12.9%	9.3%
**5**	Translators are available during checkups and hospital visits	29.4%	39.9%	7.3%	16.1%	7.3%

Table S3. Descriptive statistic for accessibility (n = 248).

No	Accessibility	Strongly Disagree	Disagree	Neutral	Agree	Strongly Agree
1	It is easy to travel to health facilities.	0.8%	2.0%	3.2%	61.7%	32.3%
2	I do not have problems of being unable to pay for the cost of travel.	1.2%	7.3%	15.7%	58.1%	17.7%
3	Distance from residence to hospital is not too far	0%	3.6%	7.3%	69.8%	19.4%
4	Emergency van is always available upon request for serious condition.	0.8%	6.5%	12.1%	56%	24%

Table S4. Descriptive statistic for accommodation (n = 248).

No	Accommodation	Strongly Disagree	Disagree	Neutral	Agree	Strongly Agree
**1**	I have ever received expedited services during my visits to the hospital.	3.6%	21.4%	15.7%	33.1%	26.2%
**2**	I have received help from volunteers at the hospital	1.2%	23.8%	14.5%	41.5%	19%
**3**	I feel comfortable receiving health services.	0%	4.4%	11.7%	62%	21.8%
**4**	I received health service guidelines.	0.4%	19 %	12.1%	47.6%	21%
**5**	The guideline is easy to understand.	0.4%	16.5%	15.7%	43.1%	24.2%

Table S5. Descriptive statistic for affordability.

No	Affordability	Strongly Disagree	Disagree	Neutral	Agree	Strongly Agree
1	I had no difficulties getting national health insurance.	0.4%	2.4%	8.9%	58.5%	29.8%
2	I had to pay out of pocket in addition to the regular copayment and registration fee.	1.6%	12.5%	15.3%	53.2%	17.3%
3	I have enough money to pay out of pocket.	3.2%	10.1%	21.8%	41.1%	23.8%
4	The service charge is affordable for me.	1.2%	2.8%	10.5%	53.6%	31.9%
5	I feel comfortable to pay extra for medicine/health services if needed.	6.5%	7.7%	16.5%	52.4%	16.9%

Table S6. Descriptive statistic for acceptability.

No	Acceptability	Strongly Disagree	Disagree	Neutral	Agree	Strongly Agree
1	When visiting a health facility, the health workers respect me.	1.2%	1.2%	8.1%	59.3%	30.2%
2	The health workers pay attention to my needs.	0%	3.2%	8.5%	61.3%	27%
3	The physician visits me thoroughly.	0.4%	4.4%	12.1%	5.2%	27.8%
4	I have trust in health workers.	0%	4.0 %	12.1%	59.7%	24.2%
5	I feel like receive equal health services to locals.	0%	0.8%	11.3%	58.5%	29.4%
